# Oxidative Stress and Mitochondrial Complex I Dysfunction Correlate with Neurodegeneration in an α-Synucleinopathy Animal Model

**DOI:** 10.3390/ijms231911394

**Published:** 2022-09-27

**Authors:** Adriana Morales-Martínez, Paola A. Martínez-Gómez, Daniel Martinez-Fong, Marcos M. Villegas-Rojas, Francisca Pérez-Severiano, Miguel A. Del Toro-Colín, Karen M. Delgado-Minjares, Víctor Manuel Blanco-Alvarez, Bertha Alicia Leon-Chavez, Omar Emiliano Aparicio-Trejo, Mauricio T. Baéz-Cortés, Maria-del-Carmen Cardenas-Aguayo, José Luna-Muñoz, Mar Pacheco-Herrero, Quetzalli D. Angeles-López, Irma A. Martínez-Dávila, Citlaltepetl Salinas-Lara, José Pablo Romero-López, Carlos Sánchez-Garibay, Adolfo R. Méndez-Cruz, Luis O. Soto-Rojas

**Affiliations:** 1 Laboratorio de Neurofarmacología Molecular y Nanotecnología, Instituto Nacional de Neurología y Neurocirugía Manuel Velasco Suárez, Mexico City 14269, Mexico; 2 Laboratorio de Patogenesis Molecular, Laboratorio 4, Edificio A4, Carrera Médico Cirujano, Facultad de Estudios Superiores Iztacala, Universidad Nacional Autónoma de México, Tlalnepantla 54090, Mexico; 3 Sección de Estudios de Posgrado e Investigación, Escuela Superior de Medicina, Instituto Politécnico Nacional, Mexico City 11340, Mexico; 4 Red de Medicina para la Educación y Desarrollo y la Investigación Científica de Iztacala (Red MEDICI), Carrera Médico Cirujano, Facultad de Estudios Superiores Iztacala, Universidad Nacional Autónoma de México, Tlalnepantla 54090, Mexico; 5 Departamento de Fisiología, Biofísica y Neurociencias, Centro de Investigación y de Estudios Avanzados del IPN, Mexico City 07360, Mexico; 6 Unidad Profesional Interdisciplinaria de Biotecnología del Instituto Politécnico Nacional (UPIBI-IPN), Mexico City 07340, Mexico; 7 Facultad de Enfermería, Benemérita Universidad Autónoma de Puebla, Av. 25 Poniente 1304, Los Volcanes, Puebla 72410, Mexico; 8 Facultad de Ciencias Químicas, Benemérita Universidad Autónoma de Puebla, Av. 14 Sur y Av. San Claudio, Cd. Universitaria, Puebla 72570, Mexico; 9 Departamento de Fisiopatología Cardio-Renal, Instituto Nacional de Cardiología “Ignacio Chávez”, Mexico City 14080, Mexico; 10 Laboratory of Cellular Reprogramming, Departamento de Fisiología, Facultad de Medicina, Universidad Nacional Autónoma de México, Av. Universidad No. 3000, Coyoacán, Mexico City 04510, Mexico; 11 National Dementia BioBank, Ciencias Biológicas, Facultad de Estudios Superiores Cuautitlán, Universidad Nacional Autónoma de México, México City 53150, Mexico; 12 National Brain Bank, Universidad Nacional Pedro Henríquez Ureña, Santo Domingo 11805, Dominican Republic; 13 Neuroscience Research Laboratory, Faculty of Health Sciences, Pontificia Universidad Católica Madre y Maestra, Santiago de los Caballeros 51000, Dominican Republic; 14 Facultad de Estudios Superiores Cuautitlán, Universidad Nacional Autónoma de México, Cuautitlán Izcalli 54740, Mexico; 15 Departamento de Neuropatología, Instituto Nacional de Neurología y Neurocirugía Manuel Velasco Suárez, Mexico City 14269, Mexico; 16 Laboratorio de Inmunología UMF, Facultad de Estudios Superiores Iztacala, Universidad Nacional Autónoma de México, Tlalnepantla 54090, Mexico

**Keywords:** Parkinson’s disease, α-synucleinopathy, mitochondrial dysfunction, BSSG, oxidative stress, nitrosative stress

## Abstract

The α-synucleinopathies constitute a subset of neurodegenerative disorders, of which Parkinson’s disease (PD) is the most common worldwide, characterized by the accumulation of misfolded α-synuclein in the cytoplasm of neurons, which spreads in a prion-like manner to anatomically interconnected brain areas. However, it is not clear how α-synucleinopathy triggers neurodegeneration. We recently developed a rat model through a single intranigral administration of the neurotoxic β-sitosterol β-D-glucoside (BSSG), which produces α-synucleinopathy. In this model, we aimed to evaluate the temporal pattern of levels in oxidative and nitrosative stress and mitochondrial complex I (CI) dysfunction and how these biochemical parameters are associated with neurodegeneration in different brain areas with α-synucleinopathy (*Substantia nigra pars compacta*, the striatum, in the hippocampus and the olfactory bulb, where α-syn aggregation spreads). Interestingly, an increase in oxidative stress and mitochondrial CI dysfunction accompanied neurodegeneration in those brain regions. Furthermore, in silico analysis suggests a high-affinity binding site for BSSG with peroxisome proliferator-activated receptors (PPAR) alpha (PPAR-α) and gamma (PPAR-γ). These findings will contribute to elucidating the pathophysiological mechanisms associated with α-synucleinopathies and lead to the identification of new early biomarkers and therapeutic targets.

## 1. Introduction

Parkinson’s disease (PD) is the most common motor neurodegenerative disorder worldwide [1], known for its motor impairments such as rest tremor, rigidity, bradykinesia, and postural instability. Nonmotor alterations, including hyposmia, depression, and cognitive dysfunction, precede motor deficits [2,3]. Increasing evidence has linked misfolded α-synuclein (α-syn) to PD neurodegeneration and consequently to the motor and nonmotor manifestations [3,4,5]. Likewise, widespread α-synucleinopathy has been demonstrated in the nigrostriatal dopaminergic system and other nondopaminergic brain regions of the peripheral and autonomic nervous systems [6,7,8]. The α-synucleinopathies are a group of neurodegenerative disorders, with PD representing the most common phenotype, in which the pathognomonic hallmark is the intracytoplasmic accumulation of misfolded α-syn, mainly in neurons and, in some instances, in glial cells [9,10,11]. In addition, pathological α-syn has been postulated to spread in a prion-like manner to neuroanatomically interconnected regions [3,4,9]. However, the mechanisms by which misfolding and propagation of α-syn cause neurodegeneration in various brain regions in PD and other α-synucleinopathies remain unclear.

Oxidative/nitrosative stress and mitochondrial dysfunction are involved in PD neurodegeneration and other α-synucleinopathies [12,13,14,15,16]. Oxidative stress is characterized by the excessive production of reactive oxygen species (ROS), which can cause damage by reacting with lipids (lipid peroxidation; LP), proteins, and nucleic acids [17]. Similarly, in nitrosative stress, reactive nitrogen species (RNS) can promote LP and trigger harmful effects by affecting cellular respiration [18,19]. Furthermore, during the LP process, the formation of neurotoxic metabolites triggers positive feedback exacerbating the oxidative/nitrosative damage and apoptosis [12,20,21]. Moreover, an association between neurodegeneration and mitochondrial dysfunction has been observed in brains and animal models of PD [22,23]. Interestingly, in vitro and in vivo assays have shown that α-syn can lead to mitochondrial damage through the following mechanisms [24,25,26,27]: (i) inhibition of complex I (CI); (ii) increased release of cytochrome c; (iii) altered homeostasis of calcium and iron; (iv) increased production of nitric oxide; and (v) increased levels of mitochondrial turnover, specifically, an event called mitophagy.

Multiple animal models exist for the study of PD; however, only a few of them develop both α-synucleinopathy and neurodegeneration. For example, some animal models induced by neurotoxins develop motor alterations, oxidative stress, and mitochondrial dysfunction [28,29,30], but lack α-synucleinopathy [31]. Furthermore, some of these models fail to mimic progressive and chronic neuronal death and, therefore, are unsuitable for developing neuroprotective and neuroregenerative therapies [31,32]. Furthermore, the genetically modified animal models produce pathological α-syn aggregates, which are rarely associated with a representative loss of the neuronal population and progressive motor disabilities [31,32,33,34]. Moreover, in these models, other brain regions have not evaluated the pathological α-syn aggregation and, consequently, their association with alterations in motor and nonmotor behaviors has not been reported [35,36,37,38].

Recently, a rat PD model was developed by an oral or local (intranigral) administration of the neurotoxic β-sitosterol β-D-glucoside (BSSG), which generates pathological α-syn aggregates and neurodegeneration of the nigrostriatal system [4,31,39,40,41]. Remarkably, our work group demonstrated that the intranigral administration of BSSG first produces intracytoplasmic deposits of pathological α-syn aggregates in a few neurons that progressively invade more neurons in the injured and contralateral *substantia nigra pars compacta* (SNpc) and later spread to several interconnected brain areas in a prion-like manner [4]. In this model, α-synucleinopathy occurs in the following midbrain nuclei and brain regions: SNpc, red nucleus, *substantia nigra pars reticulata*, ventral tegmental area, striatum, motor cortex, hippocampus, and olfactory bulb (OB), among other brain areas [4]. The spreading of these pathological α-syn aggregates could lead to motor and nonmotor alterations similar to those of PD patients. Therefore, the intranigral administration of BSSG is a more suitable animal model for PD α-synucleinopathy [3,40]. However, it is unknown whether the BSSG-induced PD model develops oxidative/nitrosative stress, mitochondrial dysfunction, and neurodegeneration in the brain areas where the α-syn aggregates are seeded. Therefore, the present study aimed to demonstrate whether a single intranigral BSSG administration triggers the neuropathological events mentioned above in the four previously described brain areas. Our results in vivo showed an increase in the production of ROS and LP, a decrease in the activity of the mitochondrial CI in the four brain regions evaluated, and an increase in the RNS, specifically in the nigrostriatal system. Interestingly, these events were accompanied by a decrease in the neuronal population of the brain regions studied. In addition, in silico assays suggest that the neurotoxic BSSG can bind with high-affinity to peroxisome proliferator-activated receptors (PPAR) alpha (PPAR-α) and gamma (PPAR-γ), closely implicated in the oxidative stress response and mitochondrial dysfunction. These results suggest that oxidative damage and mitochondrial CI dysfunction contribute to neurodegeneration in this model of α-synucleinopathy. Therefore, understanding the pathophysiological mechanisms associated with neurodegeneration will allow the validation of new early biomarkers and timely therapies for α-synucleinopathies.

## 2. Results

### 2.1. Oxidative Damage Was Detected in Several Brain Areas following Intranigral BSSG Administration

**Figure 1:**
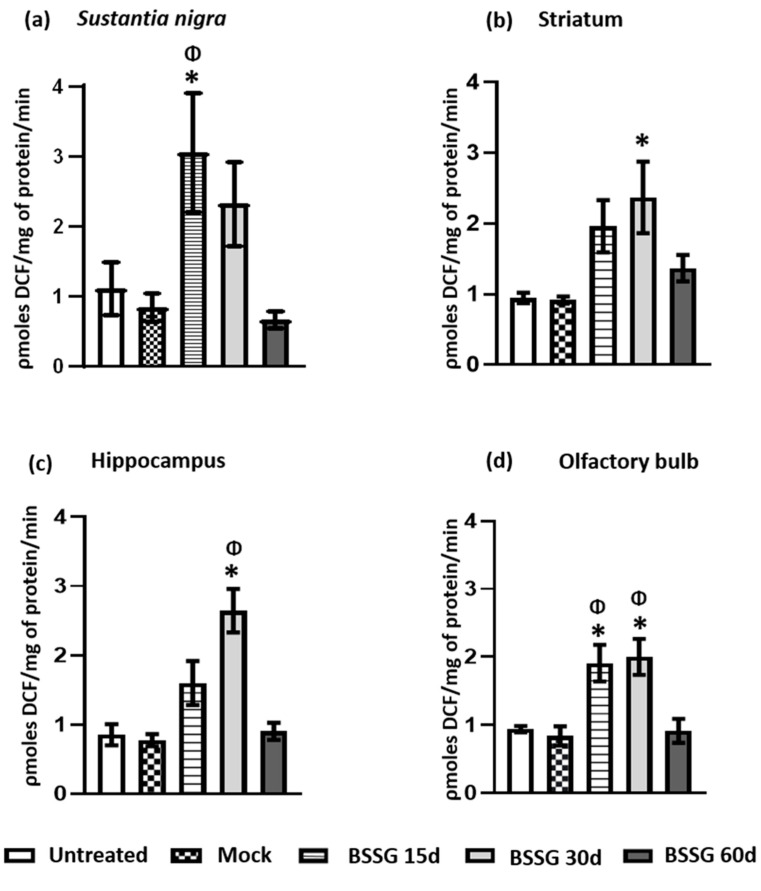
Intranigral administration of BSSG causes ROS production at different time points in several brain regions. The determination of ROS production was evaluated on the ipsilateral side of BSSG administration in the *substantia nigra* (**a**), striatum (**b**), hippocampus (**c**), and olfactory bulb (**d**). Statistical analysis was conducted by one-way ANOVA, followed by Tukey’s post hoc test. The values plotted are the mean ± SEM calculated from the measurements of independent rats (*n* = 6 animals) per subgroup. The asterisk * indicates *p* < 0.05 when the BSSG subgroups were compared to the mock group. The symbol Φ indicates *p* < 0.05 when the BSSG 60d was compared to the other BSSG subgroups.

**Figure 2:**
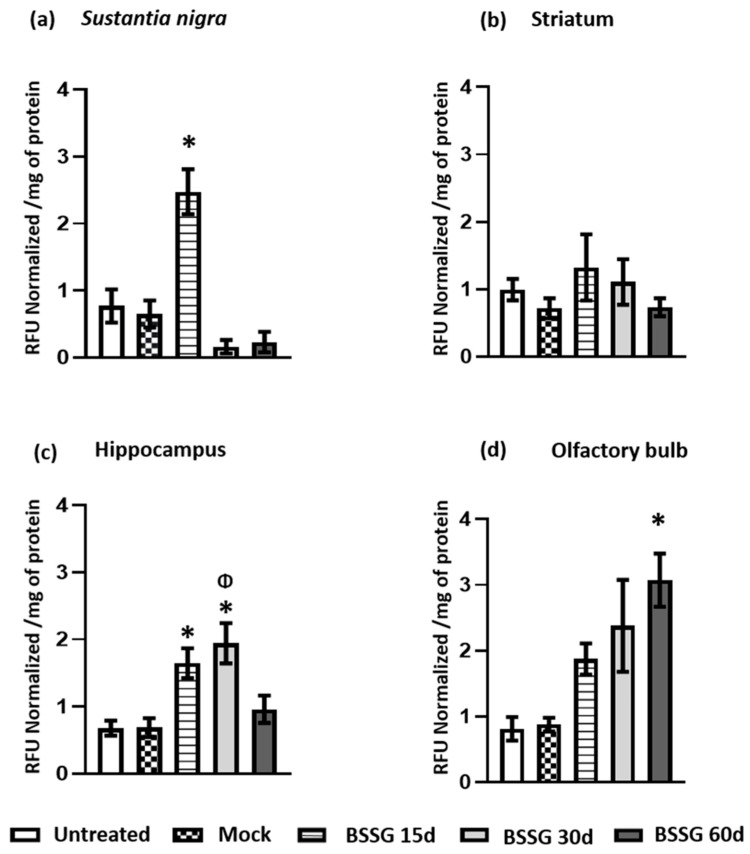
Unilateral intranigral BSSG administration results in LP observed at different times depending on the brain area analyzed. LP was evaluated in the *substantia nigra* (**a**), striatum (**b**), hippocampus (**c**), and olfactory bulb (**d**). One-way ANOVA, followed by Tukey’s post hoc test, was used for comparisons between groups. The values are plotted to represent the mean ± SEM calculated from the measurements of samples derived from independent rats (*n* = 6 animals per subgroup). The asterisk * indicates *p* < 0.05 when the BSSG subgroups were compared to the mock group. The symbol Φ indicates *p* < 0.05 when the BSSG 30d was compared to the BSSG 60d in (**c**).

Oxidative stress in brain tissue was assessed by measuring ROS (Figure 1) and LP (Figure 2). In the four brain regions evaluated, no statistically significant differences were observed between the intact and mock groups (*p* > 0.05; Figure 1 and Figure 2). The results are therefore always presented below compared to the mock group.

The intranigral BSSG administration caused a three-fold increase (267%; *p* < 0.05) in ROS production in the *substantia nigra* (SN), i.e., the site of injection, 15 days after the BSSG injury compared with the mock group (Figure 1a). At 30 days, ROS production started to decline (173% above control, *p* > 0.05), and by day 60, ROS production had returned to the basal level (Figure 1a). LP in this nucleus was evidenced by a significant increase (281%; *p* < 0.05) at the same time (BSSG 15d) compared with the mock group (Figure 2a). The results suggest that ROS production and LP are present at the site of BSSG injection 15 days after the insult.

The same intranigral BSSG administration triggered a delayed and more modest increase in ROS production in the striatum on day 30 (157%; *p* < 0.05; Figure 1b), which was not matched by increased LP (*p* > 0.05; Figure 2b). A similar increase was also observed in the hippocampus, where an elevation in ROS production was also observed on day 30 (241%; *p* < 0.05) compared with the mock group (Figure 1c). In the hippocampus, a statistically significant increase in LP was observed on days 15 (138%; *p* < 0.05) and 30 (181%; *p* < 0.05) compared with the mock group (Figure 2c). Notably, the SN, striatum, and hippocampus showed no ROS production or LP increase 60 days post-BSSG administration (Figure 1 and Figure 2; *p* > 0.05).

A different pattern was observed in the OB (Figure 1d and Figure 2d). The unilateral and intranigral BSSG administration caused an ROS elevation in the OB on days 15 (127%; *p* < 0.05) and 30 (138%; *p* < 0.05), returning to baseline values compared with the mock group at day 60 (Figure 1d). Interestingly, in this cerebral area, we observed a progressive increase in LP on days 15 (113%; *p* > 0.05), 30 (170%; *p* > 0.05), and 60 (249%; *p* < 0.05) compared with the mock group (Figure 2d).

### 2.2. Intranigral BSSG Administration Causes a Time-Delayed Nitrosative Response in the Nigrostriatal Pathway

**Figure 3:**
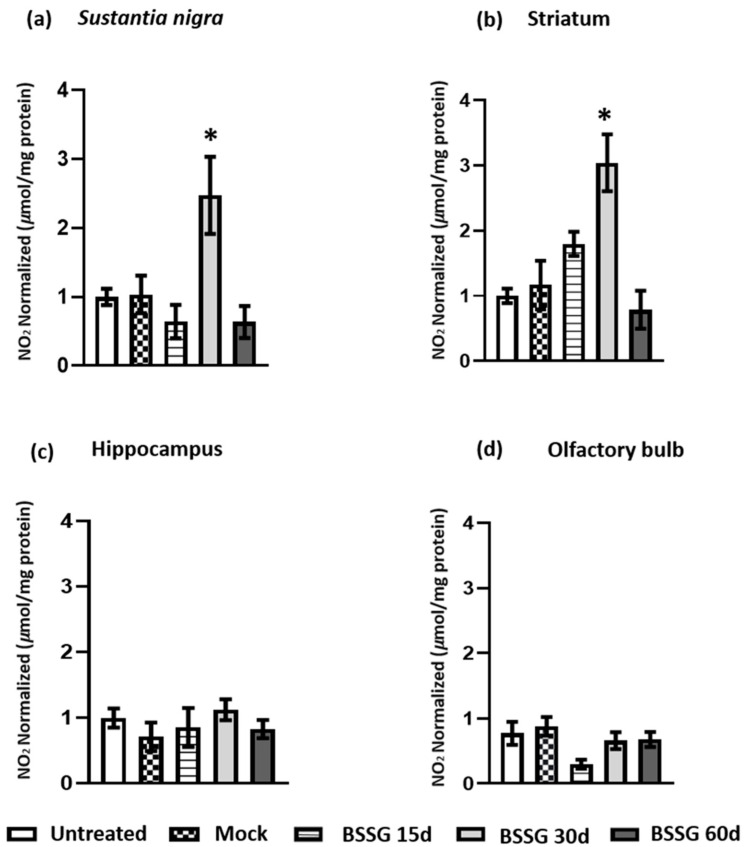
A single intranigral BSSG administration triggers time-delayed nitrosative stress in the nigrostriatal areas. The determination of nitrites levels was on the ipsilateral side of the administration of BSSG in the *substantia nigra* (**a**), striatum (**b**), hippocampus (**c**), and olfactory bulb (**d**). Statistics were by one-way ANOVA, followed by Tukey’s post hoc test. The values are plotted to represent the mean ± SEM calculated from the measurements of independent rats (*n* = 6 animals per subgroup). The asterisk * indicates *p* < 0.05 when the BSSG subgroups were compared to the mock group.

The nitrosative response was determined through nitrite production in the four regions described above (Figure 3). The intranigral BSSG administration caused a particular increase in nitrite production only on day 30 in the SN (239.8%; *p* < 0.05) and striatum (262%; *p* < 0.05) nuclei compared with the mock group (Figure 3a,b). We did not find a significant increase (*p* > 0.05) in nitrites in either the hippocampus nor the OB (Figure 3c,d).

### 2.3. A Single Intranigral BSSG Administration Triggers a Decrease in the Mitochondrial Complex I (CI) Activity in Several Brain Regions

**Figure 4:**
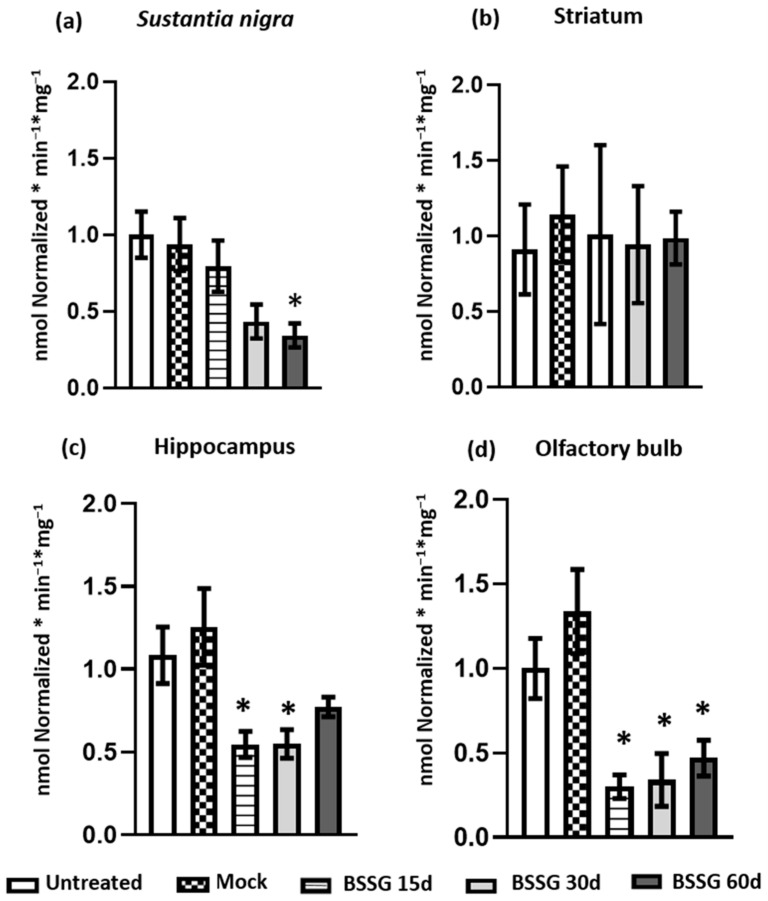
The unilateral intranigral BSSG administration causes a decrease in the mitochondrial complex I (CI) activity in different brain areas. The activity of mitochondrial CI was determined on the ipsilateral side to the administration of BSSG in the *substantia nigra* (**a**), striatum (**b**), hippocampus (**c**), and olfactory bulb (**d**). One-way ANOVA followed by Tukey’s post hoc testing was used for statistical analysis. The values are plotted to represent the mean ± SEM calculated from the measurements of independent rats (*n* = 6 animals per subgroup). An asterisk * indicates *p* < 0.05 when the BSSG subgroups were compared to the mock group.

The intranigral BSSG administration caused a progressive decrease in mitochondrial CI activity in the SN (Figure 4a) on days 15 (15.17%; *p* > 0.05), 30 (53.8%; *p* > 0.05), and 60 (63.5%; *p* < 0.05) when compared to the mock group. In contrast, mitochondrial CI activity was unaffected in the striatum (Figure 4b). Concerning the hippocampus, a decrease in CI activity was observed on days 15 (56.4%; *p* < 0.05), 30 (56.2%; *p* < 0.05), and 60 (38.3%, *p* > 0.05) compared to the mock group (Figure 4c). A similar response occurred in the OB, where the decrease in CI activity was, respectively, 77.5% on day 15 (*p* < 0.05), 74.5% on day 30 (*p* < 0.05), and 64.8% on day 60 (*p* < 0.05) after BSSG injury compared to the mock group (Figure 4d). Once again, no statistically significant differences between the intact and mock groups were observed in the cerebral regions analyzed (*p* > 0.05; Figure 4).

### 2.4. A Single Intranigral BSSG Administration Causes a Decrease in the Neuronal Population in Several Brain Regions

**Figure 5:**
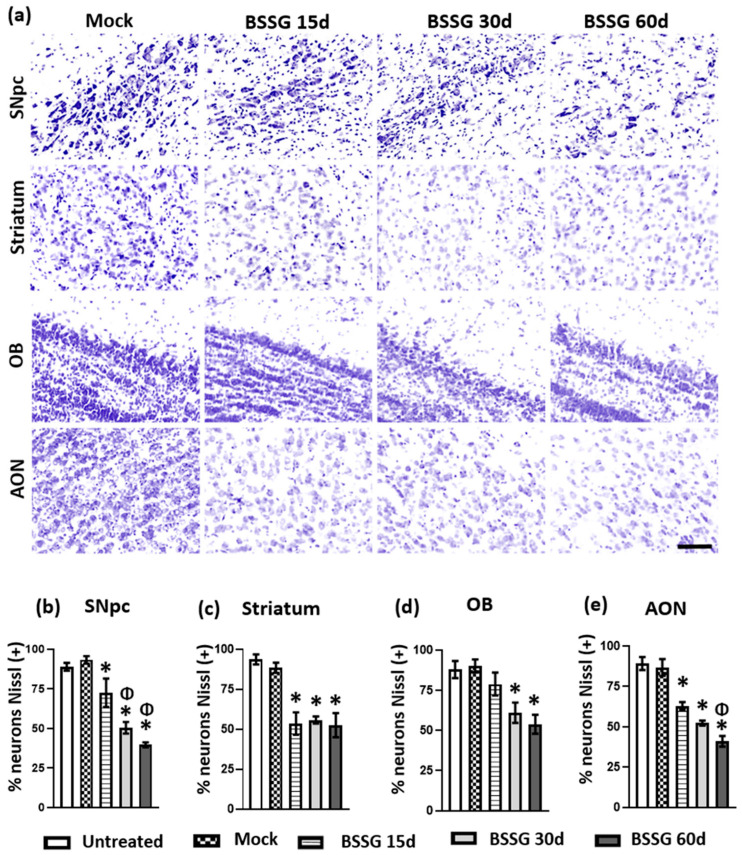
A single intranigral BSSG administration triggers a decrease in the ipsilateral neuronal population of different brain areas. Panel (**a**) shows representative Nissl staining micrographs of several subgroups (as indicated at the top of the Figure) and the respective brain regions (as indicated at the left margin of each panel row). The areas evaluated were: *substantia nigra pars compacta* (SNpc), striatum, olfactory bulb (OB), and anterior olfactory nucleus (AON). The scale bar = 50 µm is common for all micrographs. The graphs show the neuronal quantification of SNpc (**b**), striatum (**c**), OB (**d**), and AON (**e**). Statistical analysis was performed using one-way ANOVA and Tukey’s post hoc test. The values in each bar represent the mean ± SEM calculated from the measurements at three anatomical levels. *n* = 6 independent rats per subgroup. The asterisk * represents *p* < 0.05 when the BSSG subgroups were compared to the mock group, whereas the symbol Φ represents *p* < 0.05 when the BSSG 15d was compared to the other BSSG subgroups.

**Figure 6:**
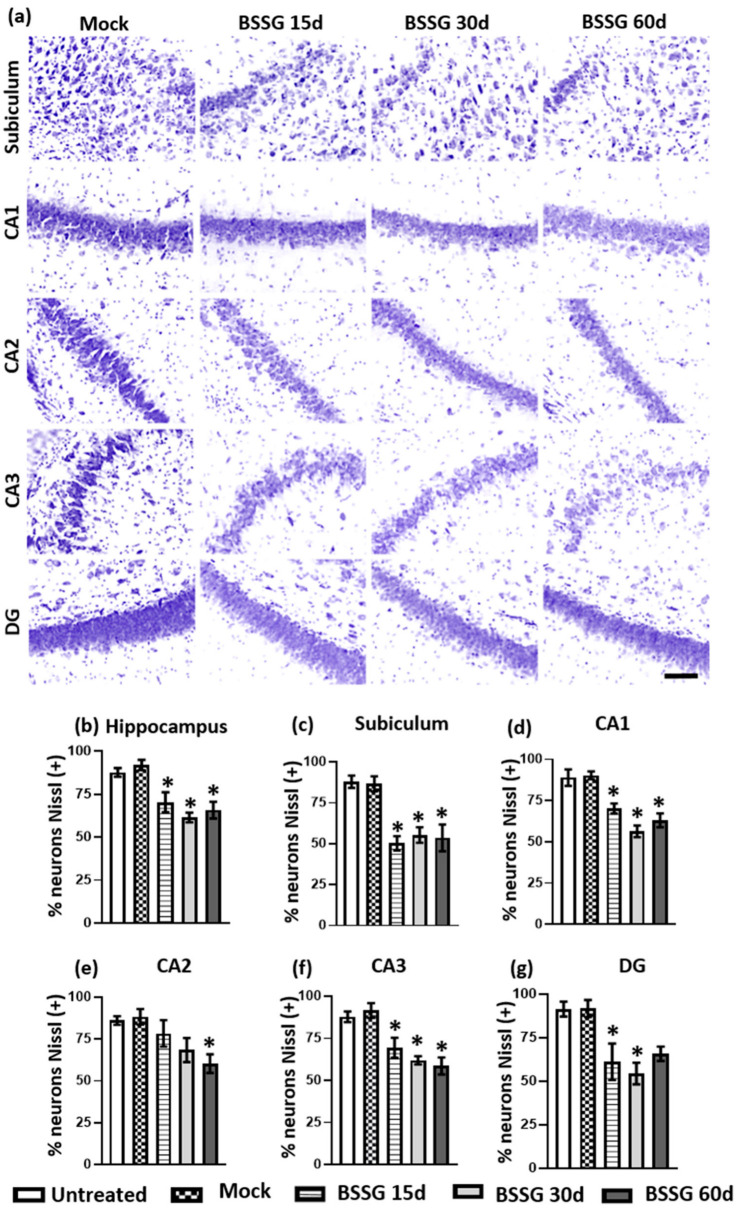
A single intranigral BSSG administration causes a decrease in the ipsilateral neuronal population of different hippocampal regions. Panel (**a**) shows representative Nissl staining micrographs of the several subgroups (as shown at the top) and hippocampal areas (as shown at the left margin of each row). The areas evaluated included the subiculum, CA1, CA2, CA3, and dentate gyrus (DG). The scale bar = 50 µm is common for all micrographs. The graphs show the neuronal quantification of the total hippocampus (**b**), subiculum (**c**), CA1 (**d**), CA2 (**e**), CA3 (**f**), and DG (**g**). One-way ANOVA, Tukey’s post hoc. The values are the mean ± SEM calculated from the measurements in three anatomical levels. *n* = 6 independent rats per subgroup. * *p* < 0.05 when the BSSG subgroups were compared to the mock group.

The neuronal population was quantified through Nissl-positive soma cells in different brain regions (Figure 5 and Figure 6). The unilateral, intranigral BSSG injection led to a significant progressive decrease in neuronal density in the SNpc (Figure 5a,b) on days 15 (20.7%; *p* < 0.05), 30 (42.8% *p* < 0.05), and 60 (53.5%; *p* < 0.05) when were compared to the mock group. A statistical difference was also present between BSSG 15d, BSSG 30d, and BSSG 60d (*p* < 0.05), substantiating the progressive nature of neurodegeneration.

A significant decrease in the neuronal population of the striatum was observed on days 15 (39.36%; *p* < 0.05), 30 (36.87% *p* < 0.05), and 60 (40.49%; *p* < 0.05) when compared to the mock group (Figure 5a,c). Likewise, progressive neuronal loss was observed in the OB on days 15 (12.43%; *p* > 0.05), 30 (32.24%; *p* < 0.05), and 60 (40.19%; *p* < 0.05) compared with the mock group (Figure 5a,d). In the anterior olfactory nucleus (AON), a progressive decrease in neuronal numbers was evidenced on days 15 (23.3%; *p* < 0.05), 30 (39.6%; *p* < 0.05), and 60 (52.7%; *p* < 0.05) after BSSG injury compared to the mock group (Figure 5a,e). Additionally, in this brain nucleus, the mean value of neuronal counts in the BSSG 15d group was statistically different compared to the BSSG 60d group (*p* < 0.05).

In addition, we found that the intranigral and unilateral BSSG administration caused a decrease in the neuron population of the hippocampus and its respective areas compared with the mock group (Figure 6): results from the entire hippocampus were, respectively, 23.6% on day 15 (*p* < 0.05), 33.2% on day 30 (*p* < 0.05), and 28.6% on day 60 (*p* < 0.05) (Figure 6a,b). When analyzed per subregions, the decrease in neuronal population was as follows: in the subiculum (Figure 6a,c), day 15 (42%; *p* < 0.05), day 30 (36.3%; *p* < 0.05), day 60 (38.2%; *p* < 0.05); in the CA1 (Figure 6a,d), day 15 (22.2%; *p* < 0.05), day 30 (37.5%; *p* < 0.05), day 60 (30.2%; *p* < 0.05); in the CA2 (Figure 6a,e), day 15 (11.3%; *p* > 0.05), day 30 (22.5%; *p* > 0.05), day 60 (31.7%; *p* < 0.05); in the CA3 (Figure 6a,f), day 15 (24.1%; *p* < 0.05), day 30 (32.3%; *p* < 0.05), day 60 (36%; *p* < 0.05); in the dentate gyrus (DG; Figure 6a,g), day 15 (33.3%; *p* < 0.05), day 30 (40.7%; *p* < 0.05), day 60 (28.3%; *p* > 0.05). In all cerebral areas evaluated, no statistically significant differences were found between the intact and mock groups (*p* > 0.05; Figure 5 and Figure 6), thus demonstrating the causal relationship between the BSSG injection and the generalized neuronal loss in the brain areas studied.

### 2.5. BSSG Has a High Affinity for PPAR-α and PPAR-γ, Targets Closely Related to Oxidative Stress and Mitochondrial Dysfunction

Molecular docking studies were carried out on four targets: PPARα, PPARγ, MAP kinase p38 alpha (MAPK14), and Histone deacetylase 6 (HDAC6). These proteins were chosen according to their participation in oxidative processes. Once the molecules were prepared, the blind docking of each protein with the BSSG molecule was performed.

**Figure 7:**
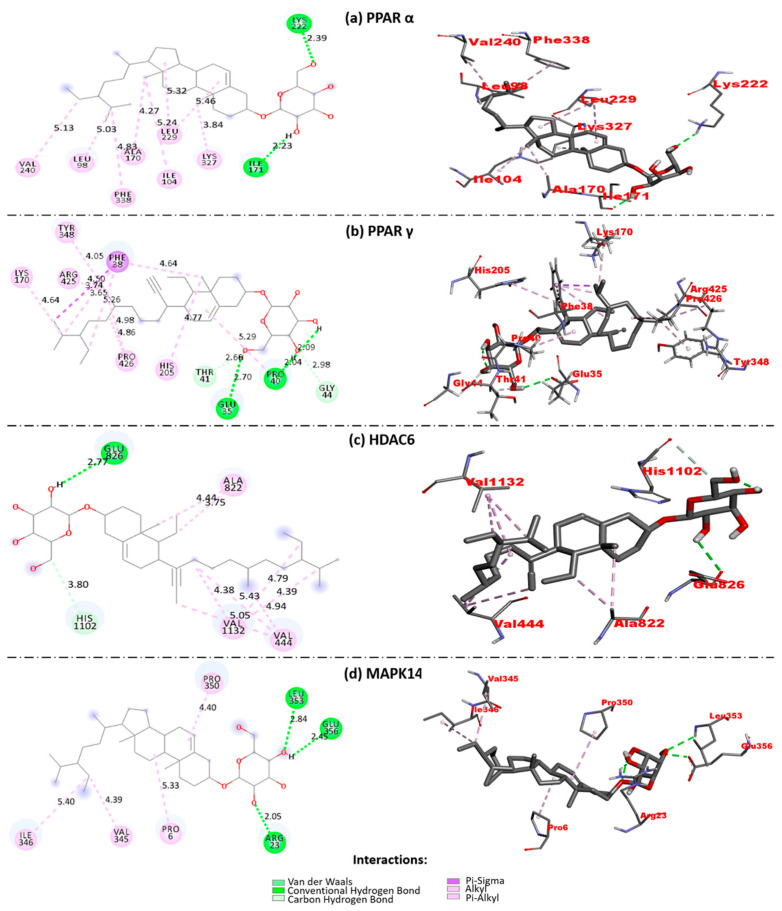
Two-dimensional (left column) and three-dimensional (right column) contact maps of molecular docking between BSSG and targets closely related to oxidative stress and mitochondrial dysfunction. BSSG has different types of interactions (represented with color codes) with peroxisome proliferator-activated receptors alpha (**a**) and gamma (**b**), histone deacetylase 6 (HDAC6 (**c**)), and kinase p38 alpha (MAPK14 (**d**)). These 2D and 3D representations were obtained from Biovia Discovery Studio.

The interactions obtained in the coupling results were the following: hydrogen bonds and Van der Waals-type interactions, Alkyl, Pi-sigma, Pi-Alkyl (Figure 7, Table 1). Generally, these are intermolecular interactions that are key to ligand stabilization. Hydrogen bonds (h-bonds), with an average distance of 2.7–3.0 Å, result from the interaction between a protein and its ligand or between residues of the same protein, provided either of them have hydrogen atoms that can be donors or acceptors [42,43]. Van der Waals forces are attractive forces between nonpolar molecules that are weak individually, but several together become significant in the ligand-receptor binding [44]. The other interactions (alkyl, pi-alkyl, and Pi-sigma) are weak and noncovalent bonds between hydrophobic pockets, so they are considered stabilizing interactions. Aromatic groups (of the ligand or amino acids such as tryptophan, phenylalanine, and tyrosine of the receptor) and alkyl groups participate in these interactions, which can be observed by overlapping atomic orbitals [45].

Binding affinity (ΔG, Kcal/mol) was obtained (Table 1). This parameter indicates the energy necessary for the protein–ligand interaction to take place. A low ΔG suggests a higher affinity, but a high ΔG signifies a less affinity. Therefore, it can be interpreted as the energy necessary for the protein–ligand interaction.

Table 1 shows the results obtained from the molecular couplings, highlighting the high affinity with some proteins such as PPARs with ΔG of −8.6 Kcal/mol for the α isoform and −8.5 Kcal/mol for the isoform γ. HDAC6 protein showed −8.3 Kcal/mol, followed by MAPK14 with −7.8 Kcal/mol. In addition, the interactions involved between the ligand and the proteins are observed, highlighting the h-bond type and hydrophobic interactions with specific amino acids. These interactions define the binding force between the BSSG with the different proteins. Figure 7 shows that h-bonds represent a minority, while alkyl and Van der Waals-type interactions are the most recurrent in a 2D contact map.

H bond-type interactions took place with the OH groups of the steroidal glycoside of the BSSG molecule. Hydrophobic interactions occur mainly based on β-sitosterol. The β-sitosterol is a sterol lipid with a stigmastane skeleton, which has a cholestane moiety with an ethyl group on the C24 carbon atom (Figure 7).

On the other hand, our results evidence that the positive controls bind to the same BSSG site with a similar affinity (Figure S1 and Table S1). Contrarily, the results of the negative control result suggest less stable affinity; moreover, the molecule does not bind to the BSSG binding site (Figure S2 and Table S1).

## 3. Discussion

This study used the single intranigral administration of the neurotoxic BSSG rat model to explore whether oxidative and nitrosative damage and mitochondrial dysfunction correlate with neurodegeneration in four brain regions. The evaluation of oxidative/nitrosative stress included the measurements of ROS, RNS, and LP. Then, mitochondrial CI activity experiments and neuronal population quantification were performed to confirm the neurotoxicity caused by BSSG in a temporal course. Regarding the process of oxidative damage, ROS reacts with polyunsaturated fatty acids (PUFAs) to convert them into free radicals [46], which initiates a chain reaction process producing LP. Just as oxidative stress leads to the LP process, nitrosative stress also has a similar effect [18,47]. Although nitric oxide (NO) plays a physiological role in the brain, it can also promote the production of mitochondrial ROS and RNS, exerting cytotoxic effects by affecting cellular respiration [19]. It is known that these radicals can lead to neuronal death through different pathways. The 4-Hydroxynonenal (4-HNE), one of the products of LP secondary to the activity of these oxidant species, can induce apoptosis through the intrinsic pathway involving the release of cytochrome C into the cytosol and the consequent activation of associated caspases [48]. Similarly, proinflammatory cytokines released by astrocytes in response to pathological α-syn aggregates can activate the caspase/cytochrome C signaling cascade [12].

On the other hand, LP mediated by iron and reduced glutathione (GSH) depletion leads to a cell death pathway defined as ferroptosis, which may also occur in PD [49,50]. Future experiments are required to determine if the BSSG model exhibits this death mechanism. In this respect, Shaw and colleagues showed that methionine sulfoximine (MSO), a byproduct of ancient industrial flour processing, caused a loss of GSH [51,52]. Interestingly, those authors also showed that BSSG-containing extracts exhibited the same pharmacological properties as MSO, leading to neuronal death, suggesting that oxidative stress was involved in these neurotoxic processes [51,53]. These pioneering observations are consistent with the findings of our study.

We also found that the decreased neuronal population in the four evaluated brain regions promoted by a single intranigral BSSG correlated with oxidative damage (Figure S3) and mitochondrial CI dysfunction (Figure S4). Starting with the SN, our results showed a significant affectation on day 15 after BSSG administration, which accounts for the sensorimotor disorders previously described [4,40]. Likewise, it has been shown that the SN is one of the most susceptible brain areas to biochemical changes and, therefore, is more vulnerable to neurodegeneration [54]. Consistently, the neuronal population shows a progressive decrease from 15 to 60 days after injury. Of interest in this nucleus, ROS production appeared to be followed by RNS production in a timeframe of weeks following contact with BSSG. LP and mitochondrial dysfunction could result from ROS or RNS, triggering the neuronal death that occurs mainly at 30 and 60 days after BSSG intranigral administration. In agreement with our results, studies in PD models and patients established mitochondrial dysfunction (mitochondrial CI inhibition and degreased ATP) as an essential role in the pathogenesis of the disease [55]. In contrast, a recent analysis of PD brains, specifically in the SNpc, showed increased mitochondrial density and increased expression of CI in dopaminergic axons [56]. The authors suggest that the neurons foster the mitochondrial population as a compensatory response to maintain ATP production and electrical excitability. It is, therefore, possible that the deficiency in mitochondrial CI activity observed in our study occurs in cell bodies of neurons that are being eliminated by cell death processes and not in active axons mediating synaptic transmission.

The SNpc sends dopaminergic projections to the striatum. In PD, neurodegeneration in the SNpc causes a lack of striatal dopamine, affecting motor control via circuit pathways of the basal ganglia [57]. We observed an increase in striatal ROS/RNS at day 30 postinjury, which is consistent with the locomotor impairment, evidenced by the gait impairment in rodents at a similar time in this model [4,40]. However, the increase in the ROS/RNS levels in the striatum was not accompanied by an increase in LP or mitochondrial CI dysfunction but rather a decrease in neuronal survival. This finding suggests that other mechanisms besides oxidative damage are responsible for the decrease in the neuronal population in this region or that ROS-sensitive neurons are eliminated between 15 and 30 days. Therefore, the surviving population is resistant to LP and mitochondrial dysfunction. On the other hand, the striatum of rodents is comprised of 95% of medium-sized spiny (MSNs) GABAergic neurons, and it has been hypothesized that in PD, the death of these neurons is through an excitotoxic mechanism. Furthermore, protective mechanisms, such as dystrophic changes in the length and number of its dendrites, may also occur to avoid such death [58]. Consistent with this hypothesis, we previously demonstrated dopaminergic denervation and a decreased density of MSN dendritic spines in the striatum using this intranigral BSSG animal model [4].

On the other hand, in α-synucleinopathies, an association of depression and cognitive impairment with hippocampal dysfunction has been observed [59,60]. In the BSSG-induced α-synucleinopathy animal model, we observed mitochondrial CI dysfunction and a decrease in the neuronal population in diverse hippocampal regions evaluated on days 15, 30, and 60, as well as maximum oxidative damage on day 30 after the injury. These results are closely associated with dysfunction in working and episodic memory and depression-like behavior, which were evident from day 30 after intranigral administration of BSSG [4,40].

Regarding the alterations seen in the OB, we found oxidative damage and dysfunction of the mitochondrial CI that accompanies the progressive decrease in the neuronal population from day 15 until day 60 after the lesion, supporting previous observations, where the development of hyposmia had been demonstrated from 15 days after the intranigral BSSG administration [4,40]. The mechanisms by which the administration of BSSG in the SN triggers such evident damage in the OB from the early stages of BSSG lesion are unclear. Since it was described that there is a direct pathway between SN and OB [61], we suggest that this damage may be due to retrograde propagation of α-synuclein or BSSG itself. This proposal is justified because several intranasally administrated drugs can affect several brain areas [62,63]. Therefore, olfactory pathway dysfunction becomes a target for identifying biomarkers and early therapeutic effects.

Finally, from in silico studies presented here, four targets (PPARα, PPARγ, MAPK14, and HDAC6) were found that interact with BSSG (Table 1). These targets were chosen according to their preferential expression in the Central Nervous System (CNS) and their relationship with oxidative damage and mitochondrial dysfunction [64,65,66,67]. Interestingly, the PPARs had a higher predicted affinity for BSSG than the rest of the proteins tested in molecular docking assays (Table 1). Activation of the PPARs is associated with mitochondrial biogenesis, production of antioxidant enzymes, and transcription of other PPARs isoforms. Furthermore, they participate in several processes, such as the beta-oxidation of fatty acids and neurotransmission [65,68]. In the CNS, some studies confirm that the activation of pan (multiple) and specific PPARs agonists generates neuroprotective effects against α-synucleinopathies and other neurodegenerative diseases such as Alzheimer’s disease (AD), Huntington’s disease (HD), and Amyotrophic Lateral Sclerosis (ALS) [64,65,69]. Therefore, these antecedents sustain the use of new therapies targeting PPARs to block the pathophysiology process in these neurodegenerative disorders [70,71]. For instance, PPAR-γ signaling stimulation has been shown to induce beneficial effects on mitochondrial function in animal models of these neurodegenerative disorders [64].

Conversely, PPARs deficiency has been associated with harmful processes, as occurs in the PPAR-α isoform in PD. PPAR-α deficiency produces α-syn accumulation in mitochondria, damaging complex I of the respiration chain [65]. Therefore, the high affinity of BSSG for PPARs might be the mechanism that triggers oxidative damage and mitochondrial dysfunction by inhibiting the physiological and neuroprotective effects of PPARs. Future work in our laboratories will aim to demonstrate whether BSSG neuropathological effects are inhibited or reduced by the therapeutic effects of PPARs agonists, both in this animal model of α-synucleinopathy and studies in vitro.

Our study provides evidence in vivo for the generation of ROS, RNS, and LP, and mitochondrial CI decrease, followed by the intranigral BSSG administration in the rat. Each brain area shows a different time pattern of appearance of the above parameters tightly involved in neurodegeneration. We have discussed the reasons for the differences observed and suggested they are also valid for human PD. Based on molecular docking analysis in silico, we propose a new hypothesis: PPARs are endogenous effectors of BSSG neurotoxicity. The α-synucleinopathy induced by the intranigral BSSG administration is a suitable model for understanding the pathophysiological mechanisms of neurodegenerative disorders included in α-synucleinopathy and identifying new early biomarkers and therapeutics.

## 4. Materials and Methods

### 4.1. Animals

The animals were handled according to the Mexican Official Standard NOM-062-ZOO-1999 technical specifications for the production, care, and use of laboratory animals. Adult male *Wistar* rats weighing between 210 and 230 g were supplied by the Biotherium of the Facultad de Estudios Superiores Iztacala-UNAM (Project I-186). The animals were kept under standard conditions of 12 h light–dark cycles at room temperature of 22 ± 2 °C and relative humidity of 60 ± 5%, with access to water and food *ad libitum*. From the total number of rodent animals used in this study (*n* = 90), the following study groups were formed (Figure S5): (1) untreated (UT, without surgery; *n* = 18); (2) mock (stereotaxic surgery and 1 µL dimethyl sulfoxide (DMSO) administration; *n* = 18); and the BSSG group (stereotaxic surgery and 6 µg BSSG/1 µL DMSO administration), further subdivided into three subgroups; (3) BSSG 15 days (*n* = 18); (4) BSSG 30 days (*n* = 18); (5) BSSG 60 (*n* = 18) days after the lesion.

### 4.2. Stereotaxic BSSG Administration

The BSSG stereotaxic administration was performed as previously reported [4,39,40]. First, animals were anesthetized using a mixture of xylazine (10 mg/kg)/ketamine (100 mg/kg) via intraperitoneal (i.p.) and placed in the stereotaxic apparatus (Stoelting, Wood Dale, IL, USA). Subsequently, the neurotoxic (BSSG) or vehicle (DMSO) were administrated according to the Paxinos atlas; the coordinates were anteroposterior (AP) +3.2 mm; mediolateral (ML) +2.1 mm; dorsoventral (DV) −6.6mm. A microperfusion pump (Stoelting, Wood Dale, IL, USA) and a 20-gauge dental needle were used to maintain a perfusion rate of 0.13 μL/min. Five minutes passed before removing the needle to ensure the complete diffusion of BSSG. Next, the wound was sutured with 00 silk, and finally, oxytetracycline and polymyxin B (Pfizer, Toluca, Mexico) was placed for infection prevention. Sensorimotor affectation was verified through the vibrissae-evoked forelimb placing test, as previously reported [4,40].

### 4.3. Biochemical Assays

The rodents were euthanized with an overdose (50 mg/kg of body weight) of pentobarbital via i.p., and cervical dislocation was performed. Subsequently, fresh brain coronal sections of 2 μm thick were made with a brain matrix after removing the meninges under cold conditions. From these sections, the brain areas of interest (SN, striatum, hippocampus, and OB) were dissected and stored at −80 °C until processing. As previously described, the brain tissue was homogenized in 3 mL of 0.9% saline solution to quantify ROS, LP, and nitrites [39,72,73,74]. In addition, the mitochondrial CI activity was measured in total homogenates of each tissue as previously described [75]. All values were normalized according to the healthy control group. Since *n* = 6 animals per group were used for biochemical assays, we performed two independent experiments (*n* = 3 animals per group and experiment).

#### 4.3.1. Determination of Reactive Oxygen Species

The determination of ROS was carried out using 2,7-dichloro dihydrofluorescein diacetate (DCFH-DA). First, 5 µL of tissue homogenate (0.9% saline solution) was incubated with TRIS-HEPES buffer (18:1; TRIS pH 7.6: HEPES solution containing 120 mM NaCl, 2.5 mM KCl, 1.2 mM NaH_2_PO_4_, 0.1 mM MgCl_2_, 5 mM NaHCO_3_, 6.0 mM glucose, 1.0 mM CaCl_2_, 10.0 mM HEPES) and 50 μL of 50 μM of DCFH-DA at 37 °C for 1 h in a microplate and shaken constantly. The fluorescence determination was performed in an FLx800^TM^ Multi-Detection Microplate Reader (BioTek Instruments, Inc., Winooski, VT, USA) at an excitation wavelength of 525 nm and an emission of 488 nm. The results were interpolated to a standard calibration curve of dichlorofluorescein (DCF) concentration and absorbance, then normalized and expressed in picomoles of DCF per milligram of protein per minute.

#### 4.3.2. Determination of Lipid Peroxidation

LP was evaluated through the formation of lipid-soluble fluorescent compounds. Firstly, 1 mL of the tissue homogenate (0.9% saline solution) was added to 4 mL of a chloroform–methanol mixture (2:1 *v*/*v*). Then, the samples were incubated at a temperature of 4 °C for 30 min in the dark. Finally, the methanol phase was removed, and the fluorescence of the chloroform phase was measured in a luminescence spectrophotometer (Perkin Elmer LS50B, Waltham, MA, USA) using 370 nm of excitation and 430 nm of emission wavelengths. The sensitivity of the spectrophotometer was adjusted to 140 fluorescence units with 0.001 mg/mL of a quinine standard prepared in 0.05 M sulfuric acid before the measurement of samples. The results were normalized and expressed as relative units of fluorescence (RUF) per milligram of protein.

#### 4.3.3. Determination of Nitrites

The NO production was assessed by the accumulation of nitrites (NO_2_^−^) in the supernatants of homogenates. The nitrite concentration in 10 μL of supernatant was measured using a colorimetric reaction generated by adding 10 μL of Griess reagent, composed of equal volumes of 0.1% N-(1-naphthyl) ethylenediamine dihydrochloride and 1.32% sulfanilamide in 60% acetic acid. The absorbance of the samples was determined at 540 nm with a Nanodrop (Thermo Fisher Scientific, Wilmington, NC, USA) and interpolated by using a standard curve of NaNO_2_ (1 to 10 μM) to calculate the nitrate content. The protein content was measured in the supernatants of homogenates using the Bradford method and bovine serum albumin (BSA) for the standard curve following the manufacturer’s protocol (a SmartSpec 3000 spectrophotometer; Bio-Rad, Hercules, CA, USA). The nitrite content values were expressed as normalized values.

#### 4.3.4. Mitochondrial Complex I (CI) Activity Determination in the Total Homogenate

Each tissue was homogenated in phosphate-buffered saline (PBS) pH 7.4, samples were centrifuged at 2000× *g* × 5 min, and the supernatants were used to carry out the determinations. Briefly, the measurement of CI activity was based on its capacity to oxidize nicotinamide adenine dinucleotide + hydrogen (NADH) while reducing decylubiquinone (Dub) to dihydro-decylubiquinone (DUbH2), which is then oxidized by 2,6-Dichloroindophenol (DCPIP). This oxidized product absorbs at 600 nm. Meanwhile, 20 mM rotenone was added as a specific inhibitor of CI. All the absorbance measurements were performed at 37 °C using a Synergy-Biotek (Biotek Instruments, Winooski, VT, USA) microplate reader. The specific activity of CI was determined by subtracting the activity in the presence of the inhibitor rotenone from the noninhibited activity and expressed as nmol/min/mg protein (these data were normalized by mean values of the untreated group).

### 4.4. Neuronal Quantification

For histological analysis, the animals were euthanized using an overdose (50 mg/kg of body weight, i.p.) of pentobarbital and were subsequently perfused intracardially with 100 mL of PBS, followed by 100 mL of 4% paraformaldehyde (PFA) in PBS, as previously described [4,39,73]. The brain was removed and maintained in PFA 4% for 24 h and then in 30% sucrose in PBS at 4 °C for 48 h. Subsequently, the brain was frozen and sectioned at the sagittal plane at 30 μm thickness using a sliding cryostat (Leica CM 1510S, Heidelberg, Germany). The slices were consecutively collected in 6 wells containing a tissue collection solution (TCS; 0.2 M PBS, ethylene glycol, and glycerol) at −20 °C. The tissues were dyed with 0.1% cresyl violet (Sigma-Aldrich, St. Louis, MO, USA) and mounted on slides using Entellan resin (Merck, KGaA, Darmstadt, Germany) and observed with a 20× objective of light Eclipse E400 microscope (Nikon Systems Inc., Tokyo, Japan). Neuronal quantification was analyzed with the ImageJ-win 64 software (National Institute of Health, Bethesda, MD, USA) and performed in a random area delimited at 100 µm^2^, at three anatomical levels (which were averaged) of each region per rat (*n* = 6 independent rats per subgroup), of the following brain areas: (1) *substantia nigra pars compacta* (SNpc); (2) dorsal striatum; (3) the hippocampus, which was analyzed by areas (subiculum, CA1, CA2, CA3, and DG; and (4) the olfactory pathway, in which we evaluated the granular layer of the OB and the AOB since it is the initial of odor cortex recipient of information from the OB and the target of spatiotemporal cues from the hippocampus [76]. The histological assays were also performed and analyzed by two independent experimenters.

### 4.5. Statistical Analysis

All values were normalized and analyzed using the parametric statistical test of one-way analysis of variance (ANOVA), and pairwise comparisons between groups were performed using the Tukey post hoc test analysis. Finally, results were processed, and graph construction was performed using PRISMA 8 Software, where statistical significance was considered at least when *p* ≤ 0.05. Pearson’s correlation coefficient and subsequent linear regression were determined for correlation analysis, including the control group (Mock) and the time points with the highest oxidative stress and mitochondrial dysfunction after the BSSG administration (15 and 30 days). Statistical difference was considered at *p* < 0.05.

### 4.6. In Silico Studies

#### 4.6.1. Potential Targets of BSSG

A search for potential targets for BSSG in the Swiss Target predictor server (http://www.swisstargetprediction.ch/, accessed on 4 April 2022) [77,78] identified one hundred possible targets for binding to BSSG. Then, a further refinement search through PubMed (https://pubmed.ncbi.nlm.nih.gov/, accessed on 11 April 2022) found four potential targets related to the oxidative damage processes: PPAR-α, PPAR- γ, HDAC 6, and MAPK14.

#### 4.6.2. Molecular Docking

##### Ligand and Proteins Preparation

For ligand: The BSSG molecule was built on the MolView server (https://molview.org/, accessed on 25 April 2022) from its canonical sequence obtained from PubChem (https://pubchem.ncbi.nlm.nih.gov/, accessed on 25 April 2022). Subsequently, energy minimization was performed in the Avogadro 1.5.6 software [79], using the MMFF94 force field. Gasteiger charges were added.

For proteins: From the UniProt complete rat sequences [80], structure predictions were performed for all proteins. The template sequences for PPAR γ (ID:3E00), PPAR α (ID: 2REW), HDAC6 (ID: 5G01), and MAPK14 (ID: 6YX4) were retrieved from the protein data bank (PDB). Since these template sequences are very similar to the rat sequences, we considered that performing the coupling with these models would not affect the bonding affinity. However, the pdbs of the templates do not contain the complete structure. In some cases, more than half of the amino acids are missing. For this reason, the homology prediction of the complete sequence structures was carried out in the Rosetta software [81,82], and the identity of the templates with the rat sequence of each protein was confirmed in the Swiss Model server (https://swissmodel.expasy.org, accessed on 9 May 2022). Prediction results were validated in Molprobity. In addition, a model structural alignment was carried out to calculate the root mean square deviation (RMSD) in the Pymol viewer [83]. The lowest value model of RMSD was chosen and minimized in Gromacs 2022.2 [84] with Charmm36m force field. The system for minimization was built in CHARMM-GUI [85,86], where the protein was placed in the TIP3 solution. Hydrogens were added to the structure corresponding to pH 7.4 in the PDB2PQR server [87]. Finally, pdbqt files for the ligand and the targets were obtained in AutoDock tools [88].

##### Protein–Ligand Interaction

Because some structural changes were found near the ligand binding site of the molecules used, it was decided to perform blind docking. The BSSG molecule was coupled to each target (protein) in the AutoDock Vina program [89]. After preparing the protein and ligand, the interaction grids were produced in AutoDock tools. The size of grid boxes (coordinates x, y, z) was different for each protein, according to the volume of each one. Blind docking was performed so the program could search for binding sites on the surfaces of complete proteins choosing an exhaustiveness of 12. Free energies of affinity of the best docking pose were reported. Furthermore, the Discovery Studio program was also used to report interactions between ligand–target in the 2D and 3D contact maps [90].

##### Positive and Negative Controls

The molecular docking assays were validated by comparing the values of BSSG with respective positive and negative controls, as reported previously [91]. The positive controls were two molecules structurally similar to BSSG and were obtained from PubChem (https://pubchem.ncbi.nlm.nih.gov/, accessed on 29 August 2022). For the negative control, a molecule was randomly selected from PubChem which was structurally different from BSSG. Then, the molecules were coupled with each protein target (PPAR α, PPAR γ, HDAC6, and MAPK14) using the corresponding grid box coordinates. Next, control preparation and molecular docking were performed as described above. Finally, the 3D representations were obtained from AutoDock tools [88].

## Figures and Tables

**Table 1:** Interactions of molecular docking of BSSG to several targets associated with oxidative damage and mitochondrial dysfunction.

Molecular Docking for BSSG as the Ligand			
Protein	Binding Energy ΔG (Kcal/mol)	Interaction Residues	Interaction Type
**PPAR α**	−8.6	Lys 222, Ile 171	H-bond
		Lys 327, Val 240, Ala 170, Leu 229, Ile 104, Leu 98, Phe 338	Alkyl
**PPAR γ**	−8.5	Asn 206, Pro 40, Gly 44, Glu 35	H-bond
		Lys70, Pro426, Tyr 348, Thr34	Van der Waals
		Phe38	Pi-sigma
		His 205, Pro 426, Arg 425, Lys 170, Tyr 348.	Pi-Alkyl. Alkyl
**HDAC6**	−8.3	Glu 826, His 1102	H-bond
		Asn 1100, Ala 1101, Val 1104, Ser 825, Tyr 1098, Ser 692, Ser 693, Gln 829, Tyr 1094	Van der Waals
		Val 1132, Val 444, Ala 822	Alkyl
**MAPK14**	−7.8	Glu 356, Leu 353, Arg 23	H-bond
		Pro 350, Pro 6, Val 345, Ile 346	Alkyl

## Data Availability

The data that support the findings of this study are available from the corresponding author upon reasonable request.

## Supplementary Materials

The following supporting information can be downloaded at: https://www.mdpi.com/article/10.3390/ijms231911394/s1.

## References

[1] Balestrino R., Schapira A.H.V. (2020). Parkinson disease. Eur. J. Neurol..

[2] Lees A.J., Hardy J., Revesz T. (2009). Parkinson’s disease. Lancet.

[3] Braak H., Del Tredici K., Rub U., de Vos R.A., Jansen Steur E.N., Braak E. (2003). Staging of brain pathology related to sporadic Parkinson’s disease. Neurobiol. Aging.

[4] Soto-Rojas L.O., Martinez-Davila I.A., Luna-Herrera C., Gutierrez-Castillo M.E., Lopez-Salas F.E., Gatica-Garcia B., Soto-Rodriguez G., Bringas Tobon M.E., Flores G., Padilla-Viveros A. (2020). Unilateral intranigral administration of beta-sitosterol beta-D-glucoside triggers pathological alpha-synuclein spreading and bilateral nigrostriatal dopaminergic neurodegeneration in the rat. Acta Neuropathol. Commun..

[5] Choi M.L., Gandhi S. (2018). Crucial role of protein oligomerization in the pathogenesis of Alzheimer’s and Parkinson’s diseases. FEBS J..

[6] Wakabayashi K., Miki Y. (2018). Multi-Organ Distribution of Alpha-Synuclein Pathology in Dementia with Lewy Bodies. Brain Nerve.

[7] Beach T.G., Adler C.H., Sue L.I., Vedders L., Lue L., White Iii C.L., Akiyama H., Caviness J.N., Shill H.A., Sabbagh M.N. (2010). Multi-organ distribution of phosphorylated alpha-synuclein histopathology in subjects with Lewy body disorders. Acta Neuropathol..

[8] Braak H., Del Tredici K. (2009). Neuroanatomy and pathology of sporadic Parkinson’s disease. Adv. Anat. Embryol. Cell Biol..

[9] Bras I.C., Outeiro T.F. (2021). Alpha-Synuclein: Mechanisms of Release and Pathology Progression in Synucleinopathies. Cells.

[10] Goedert M., Jakes R., Spillantini M.G. (2017). The Synucleinopathies: Twenty Years On. J. Parkinsons Dis..

[11] Spillantini M.G., Schmidt M.L., Lee V.M., Trojanowski J.Q., Jakes R., Goedert M. (1997). Alpha-synuclein in Lewy bodies. Nature.

[12] Rizor A., Pajarillo E., Johnson J., Aschner M., Lee E. (2019). Astrocytic Oxidative/Nitrosative Stress Contributes to Parkinson’s Disease Pathogenesis: The Dual Role of Reactive Astrocytes. Antioxidants.

[13] Franco-Iborra S., Cuadros T., Parent A., Romero-Gimenez J., Vila M., Perier C. (2018). Defective mitochondrial protein import contributes to complex I-induced mitochondrial dysfunction and neurodegeneration in Parkinson’s disease. Cell Death Dis..

[14] Cubukcu H.C., Yurtdas M., Durak Z.E., Aytac B., Gunes H.N., Cokal B.G., Yoldas T.K., Durak I. (2016). Oxidative and nitrosative stress in serum of patients with Parkinson’s disease. Neurol. Sci..

[15] Schildknecht S., Gerding H.R., Karreman C., Drescher M., Lashuel H.A., Outeiro T.F., Di Monte D.A., Leist M. (2013). Oxidative and nitrative alpha-synuclein modifications and proteostatic stress: Implications for disease mechanisms and interventions in synucleinopathies. J. Neurochem..

[16] Giasson B.I., Duda J.E., Murray I.V., Chen Q., Souza J.M., Hurtig H.I., Ischiropoulos H., Trojanowski J.Q., Lee V.M. (2000). Oxidative damage linked to neurodegeneration by selective alpha-synuclein nitration in synucleinopathy lesions. Science.

[17] Dias V., Junn E., Mouradian M.M. (2013). The role of oxidative stress in Parkinson’s disease. J. Parkinsons Dis..

[18] Brown G.C., Borutaite V. (2004). Inhibition of mitochondrial respiratory complex I by nitric oxide, peroxynitrite and S-nitrosothiols. Biochim. Biophys. Acta.

[19] Cassina A., Radi R. (1996). Differential inhibitory action of nitric oxide and peroxynitrite on mitochondrial electron transport. Arch. Biochem. Biophys..

[20] Chang K.H., Chen C.M. (2020). The Role of Oxidative Stress in Parkinson’s Disease. Antioxidants.

[21] Zhang J., Perry G., Smith M.A., Robertson D., Olson S.J., Graham D.G., Montine T.J. (1999). Parkinson’s disease is associated with oxidative damage to cytoplasmic DNA and RNA in substantia nigra neurons. Am. J. Pathol..

[22] Chen J., Ren Y., Gui C., Zhao M., Wu X., Mao K., Li W., Zou F. (2018). Phosphorylation of Parkin at serine 131 by p38 MAPK promotes mitochondrial dysfunction and neuronal death in mutant A53T alpha-synuclein model of Parkinson’s disease. Cell Death Dis..

[23] Di Maio R., Barrett P.J., Hoffman E.K., Barrett C.W., Zharikov A., Borah A., Hu X., McCoy J., Chu C.T., Burton E.A. (2016). alpha-Synuclein binds to TOM20 and inhibits mitochondrial protein import in Parkinson’s disease. Sci. Transl. Med..

[24] Rocha E.M., De Miranda B., Sanders L.H. (2018). Alpha-synuclein: Pathology, mitochondrial dysfunction and neuroinflammation in Parkinson’s disease. Neurobiol. Dis..

[25] Subramaniam S.R., Chesselet M.F. (2013). Mitochondrial dysfunction and oxidative stress in Parkinson’s disease. Prog. Neurobiol..

[26] Chinta S.J., Mallajosyula J.K., Rane A., Andersen J.K. (2010). Mitochondrial alpha-synuclein accumulation impairs complex I function in dopaminergic neurons and results in increased mitophagy in vivo. Neurosci. Lett..

[27] Shavali S., Brown-Borg H.M., Ebadi M., Porter J. (2008). Mitochondrial localization of alpha-synuclein protein in alpha-synuclein overexpressing cells. Neurosci. Lett..

[28] Zhang Q.S., Heng Y., Mou Z., Huang J.Y., Yuan Y.H., Chen N.H. (2017). Reassessment of subacute MPTP-treated mice as animal model of Parkinson’s disease. Acta Pharmacol. Sin..

[29] Hernandez-Baltazar D., Zavala-Flores L.M., Villanueva-Olivo A. (2017). The 6-hydroxydopamine model and parkinsonian pathophysiology: Novel findings in an older model. Neurologia.

[30] Blum D., Torch S., Lambeng N., Nissou M., Benabid A.L., Sadoul R., Verna J.M. (2001). Molecular pathways involved in the neurotoxicity of 6-OHDA, dopamine and MPTP: Contribution to the apoptotic theory in Parkinson’s disease. Prog. Neurobiol..

[31] Van Kampen J.M., Robertson H.A. (2017). The BSSG rat model of Parkinson’s disease: Progressing towards a valid, predictive model of disease. EPMA J..

[32] Beal M.F. (2010). Parkinson’s disease: A model dilemma. Nature.

[33] Oaks A.W., Frankfurt M., Finkelstein D.I., Sidhu A. (2013). Age-dependent effects of A53T alpha-synuclein on behavior and dopaminergic function. PLoS ONE.

[34] Giasson B.I., Duda J.E., Quinn S.M., Zhang B., Trojanowski J.Q., Lee V.M. (2002). Neuronal alpha-synucleinopathy with severe movement disorder in mice expressing A53T human alpha-synuclein. Neuron.

[35] Decressac M., Kadkhodaei B., Mattsson B., Laguna A., Perlmann T., Bjorklund A. (2012). alpha-Synuclein-induced down-regulation of Nurr1 disrupts GDNF signaling in nigral dopamine neurons. Sci. Transl. Med..

[36] Koprich J.B., Johnston T.H., Reyes M.G., Sun X., Brotchie J.M. (2010). Expression of human A53T alpha-synuclein in the rat substantia nigra using a novel AAV1/2 vector produces a rapidly evolving pathology with protein aggregation, dystrophic neurite architecture and nigrostriatal degeneration with potential to model the pathology of Parkinson’s disease. Mol. Neurodegener..

[37] Lo Bianco C., Ridet J.L., Schneider B.L., Deglon N., Aebischer P. (2002). alpha-Synucleinopathy and selective dopaminergic neuron loss in a rat lentiviral-based model of Parkinson’s disease. Proc. Natl. Acad. Sci. USA.

[38] Kirik D., Rosenblad C., Burger C., Lundberg C., Johansen T.E., Muzyczka N., Mandel R.J., Bjorklund A. (2002). Parkinson-like neurodegeneration induced by targeted overexpression of alpha-synuclein in the nigrostriatal system. J. Neurosci..

[39] Luna-Herrera C., Martinez-Davila I.A., Soto-Rojas L.O., Flores-Martinez Y.M., Fernandez-Parrilla M.A., Ayala-Davila J., Leon-Chavez B.A., Soto-Rodriguez G., Blanco-Alvarez V.M., Lopez-Salas F.E. (2020). Intranigral Administration of beta-Sitosterol-beta-D-Glucoside Elicits Neurotoxic A1 Astrocyte Reactivity and Chronic Neuroinflammation in the Rat Substantia Nigra. J. Immunol. Res..

[40] Soto-Rojas L.O., Garces-Ramirez L., Luna-Herrera C., Flores-Martinez Y.M., Soto-Rodriguez G., Gatica-Garcia B., Lopez-Salas F.E., Ayala-Davila J., Gutierrez-Castillo M.E., Padilla-Viveros A. (2020). A single intranigral administration of beta-sitosterol beta-d-glucoside elicits bilateral sensorimotor and non-motor alterations in the rat. Behav. Brain Res..

[41] Van Kampen J.M., Baranowski D.C., Robertson H.A., Shaw C.A., Kay D.G. (2015). The Progressive BSSG Rat Model of Parkinson’s: Recapitulating Multiple Key Features of the Human Disease. PLoS ONE.

[42] Abelian A., Wallach J., Gaye B., Adejare A., Adejare A. (2021). Chapter 6—Pharmaceutical chemistry. Remington.

[43] Mcree D.E., Mcree D.E. (1999). Chapter 3—Computational Techniques. Practical Protein Crystallography.

[44] Schaeffer L., Wermuth C.G., Aldous S., Raboisson P., Rognan D. (2008). Chapter 14—The Role of Functional Groups in Drug–Receptor Interactions. The Practice of Medicinal Chemistry.

[45] Mecinovic J., Snyder P.W., Mirica K.A., Bai S., Mack E.T., Kwant R.L., Moustakas D.T., Heroux A., Whitesides G.M. (2011). Fluoroalkyl and alkyl chains have similar hydrophobicities in binding to the “hydrophobic wall” of carbonic anhydrase. J. Am. Chem. Soc..

[46] Nowak J.Z. (2013). Oxidative stress, polyunsaturated fatty acids-derived oxidation products and bisretinoids as potential inducers of CNS diseases: Focus on age-related macular degeneration. Pharmacol. Rep..

[47] Ortiz G.G., Morales-Sanchez E.W., Pacheco-Moises F.P., Jimenez-Gil F.J., Macias-Islas M.A., Mireles-Ramirez M.A., Gonzalez-Usigli H. (2017). Effect of melatonin administration on cyclooxygenase-2 activity, serum levels of nitric oxide metabolites, lipoperoxides and glutathione peroxidase activity in patients with Parkinson’s disease. Gac. Med. Mex..

[48] Fedorova T.N., Logvinenko A.A., Poleshchuk V.V., Muzychuk O.A., Shabalina A.A., Illarioshkin S.N. (2019). Lipid Peroxidation Products in the Blood Plasma of Patients with Parkinson’s Disease as Possible Biomarkers of Different Stages of the Disease. Neurochem. J..

[49] Wu L., Liu M., Liang J., Li N., Yang D., Cai J., Zhang Y., He Y., Chen Z., Ma T. (2021). Ferroptosis as a New Mechanism in Parkinson’s Disease Therapy Using Traditional Chinese Medicine. Front. Pharmacol..

[50] Angelova P.R., Choi M.L., Berezhnov A.V., Horrocks M.H., Hughes C.D., De S., Rodrigues M., Yapom R., Little D., Dolt K.S. (2020). Alpha synuclein aggregation drives ferroptosis: An interplay of iron, calcium and lipid peroxidation. Cell Death Differ..

[51] Shaw C.A., Bains J.S. (2002). Synergistic versus antagonistic actions of glutamate and glutathione: The role of excitotoxicity and oxidative stress in neuronal disease. Cell. Mol. Biol..

[52] Shaw C.A., Bains J.S., Pasqualotto B.A., Curry K. (1999). Methionine sulfoximine shows excitotoxic actions in rat cortical slices. Can. J. Physiol. Pharmacol..

[53] Wilson J.M., Khabazian I., Wong M.C., Seyedalikhani A., Bains J.S., Pasqualotto B.A., Williams D.E., Andersen R.J., Simpson R.J., Smith R. (2002). Behavioral and neurological correlates of ALS-parkinsonism dementia complex in adult mice fed washed cycad flour. Neuromolecular Med..

[54] Trist B.G., Hare D.J., Double K.L. (2019). Oxidative stress in the aging substantia nigra and the etiology of Parkinson’s disease. Aging Cell.

[55] Moon H.E., Paek S.H. (2015). Mitochondrial Dysfunction in Parkinson’s Disease. Exp. Neurobiol..

[56] Reeve A.K., Grady J.P., Cosgrave E.M., Bennison E., Chen C., Hepplewhite P.D., Morris C.M. (2018). Mitochondrial dysfunction within the synapses of substantia nigra neurons in Parkinson’s disease. NPJ Park. Dis..

[57] Smith Y., Villalba R.M., Raju D.V. (2009). Striatal spine plasticity in Parkinson’s disease: Pathological or not?. Parkinsonism Relat. Disord.

[58] Deutch A.Y., Colbran R.J., Winder D.J. (2007). Striatal plasticity and medium spiny neuron dendritic remodeling in parkinsonism. Park. Relat. Disord.

[59] Das T., Hwang J.J., Poston K.L. (2019). Episodic recognition memory and the hippocampus in Parkinson’s disease: A review. Cortex.

[60] Yang W., Yu S. (2017). Synucleinopathies: Common features and hippocampal manifestations. Cell Mol. Life Sci..

[61] Hoglinger G.U., Alvarez-Fischer D., Arias-Carrion O., Djufri M., Windolph A., Keber U., Borta A., Ries V., Schwarting R.K., Scheller D. (2015). A new dopaminergic nigro-olfactory projection. Acta Neuropathol..

[62] Veronesi M.C., Alhamami M., Miedema S.B., Yun Y., Ruiz-Cardozo M., Vannier M.W. (2020). Imaging of intranasal drug delivery to the brain. Am. J. Nucl. Med. Mol. Imaging.

[63] Chapman C.D., Frey W.H., Craft S., Danielyan L., Hallschmid M., Schioth H.B., Benedict C. (2013). Intranasal treatment of central nervous system dysfunction in humans. Pharm. Res..

[64] Jamwal S., Blackburn J.K., Elsworth J.D. (2021). PPARgamma/PGC1alpha signaling as a potential therapeutic target for mitochondrial biogenesis in neurodegenerative disorders. Pharmacol. Ther..

[65] Wojtowicz S., Strosznajder A.K., Jezyna M., Strosznajder J.B. (2020). The Novel Role of PPAR Alpha in the Brain: Promising Target in Therapy of Alzheimer’s Disease and Other Neurodegenerative Disorders. Neurochem. Res..

[66] Bohush A., Niewiadomska G., Filipek A. (2018). Role of Mitogen Activated Protein Kinase Signaling in Parkinson’s Disease. Int. J. Mol. Sci..

[67] Simoes-Pires C., Zwick V., Nurisso A., Schenker E., Carrupt P.A., Cuendet M. (2013). HDAC6 as a target for neurodegenerative diseases: What makes it different from the other HDACs?. Mol. Neurodegener..

[68] Hong F., Pan S., Guo Y., Xu P., Zhai Y. (2019). PPARs as Nuclear Receptors for Nutrient and Energy Metabolism. Molecules.

[69] Kiaei M. (2008). Peroxisome Proliferator-Activated Receptor-gamma in Amyotrophic Lateral Sclerosis and Huntington’s Disease. PPAR Res..

[70] Sanjay, Sharma A., Lee H.J. (2021). Role of Phytoconstituents as PPAR Agonists: Implications for Neurodegenerative Disorders. Biomedicines.

[71] Prashantha Kumar B.R., Kumar A.P., Jose J.A., Prabitha P., Yuvaraj S., Chipurupalli S., Jeyarani V., Manisha C., Banerjee S., Jeyabalan J.B. (2020). Minutes of PPAR-gamma agonism and neuroprotection. Neurochem. Int..

[72] Morales-Martinez A., Zamorano-Carrillo A., Montes S., El-Hafidi M., Sanchez-Mendoza A., Soria-Castro E., Martinez-Lazcano J.C., Martinez-Gopar P.E., Rios C., Perez-Severiano F. (2021). Rich fatty acids diet of fish and olive oils modifies membrane properties in striatal rat synaptosomes. Nutr. Neurosci..

[73] Flores-Martinez Y.M., Fernandez-Parrilla M.A., Ayala-Davila J., Reyes-Corona D., Blanco-Alvarez V.M., Soto-Rojas L.O., Luna-Herrera C., Gonzalez-Barrios J.A., Leon-Chavez B.A., Gutierrez-Castillo M.E. (2018). Acute Neuroinflammatory Response in the Substantia Nigra Pars Compacta of Rats after a Local Injection of Lipopolysaccharide. J. Immunol. Res..

[74] Perez-Severiano F., Rios C., Segovia J. (2000). Striatal oxidative damage parallels the expression of a neurological phenotype in mice transgenic for the mutation of Huntington’s disease. Brain Res..

[75] Aparicio-Trejo O.E., Reyes-Fermin L.M., Briones-Herrera A., Tapia E., Leon-Contreras J.C., Hernandez-Pando R., Sanchez-Lozada L.G., Pedraza-Chaverri J. (2019). Protective effects of N-acetyl-cysteine in mitochondria bioenergetics, oxidative stress, dynamics and S-glutathionylation alterations in acute kidney damage induced by folic acid. Free Radic. Biol. Med..

[76] Aqrabawi A.J., Kim J.C. (2020). Olfactory memory representations are stored in the anterior olfactory nucleus. Nat. Commun..

[77] Daina A., Michielin O., Zoete V. (2019). SwissTargetPrediction: Updated data and new features for efficient prediction of protein targets of small molecules. Nucleic Acids Res..

[78] Gfeller D., Michielin O., Zoete V. (2013). Shaping the interaction landscape of bioactive molecules. Bioinformatics.

[79] Hanwell M.D., Curtis D.E., Lonie D.C., Vandermeersch T., Zurek E., Hutchison G.R. (2012). Avogadro: An advanced semantic chemical editor, visualization, and analysis platform. J. Cheminform..

[80] Wang Y., Wang Q., Huang H., Huang W., Chen Y., McGarvey P.B., Wu C.H., Arighi C.N., UniProt C. (2021). A crowdsourcing open platform for literature curation in UniProt. PLoS Biol..

[81] Baek M., DiMaio F., Anishchenko I., Dauparas J., Ovchinnikov S., Lee G.R., Wang J., Cong Q., Kinch L.N., Schaeffer R.D. (2021). Accurate prediction of protein structures and interactions using a three-track neural network. Science.

[82] Song Y., DiMaio F., Wang R.Y., Kim D., Miles C., Brunette T., Thompson J., Baker D. (2013). High-resolution comparative modeling with RosettaCM. Structure.

[83] Schrödinger L., DeLano W. (2020). PyMOL. http://www.pymol.org/pymol.

[84] Pronk S., Pall S., Schulz R., Larsson P., Bjelkmar P., Apostolov R., Shirts M.R., Smith J.C., Kasson P.M., van der Spoel D. (2013). GROMACS 4.5: A high-throughput and highly parallel open source molecular simulation toolkit. Bioinformatics.

[85] Brooks B.R., Brooks C.L., Mackerell A.D., Nilsson L., Petrella R.J., Roux B., Won Y., Archontis G., Bartels C., Boresch S. (2009). CHARMM: The biomolecular simulation program. J. Comput. Chem..

[86] Jo S., Kim T., Iyer V.G., Im W. (2008). CHARMM-GUI: A web-based graphical user interface for CHARMM. J. Comput. Chem..

[87] Jurrus E., Engel D., Star K., Monson K., Brandi J., Felberg L.E., Brookes D.H., Wilson L., Chen J., Liles K. (2018). Improvements to the APBS biomolecular solvation software suite. Protein. Sci..

[88] Morris G.M., Huey R., Lindstrom W., Sanner M.F., Belew R.K., Goodsell D.S., Olson A.J. (2009). AutoDock4 and AutoDockTools4: Automated docking with selective receptor flexibility. J. Comput. Chem..

[89] Trott O., Olson A.J. (2010). AutoDock Vina: Improving the speed and accuracy of docking with a new scoring function, efficient optimization, and multithreading. J. Comput. Chem..

[90] BIOVIA Discovery Studio Dassault Systèmes BIOVIA (2020). Discovery Studio Modeling Environment.

[91] Islam M.R., Hosen M.I., Zaman A., Islam M.O. (2013). Structural, functional and molecular docking study to characterize GMI1 from Arabidopsis thaliana. Interdiscip. Sci..

