# Supplementary material for: Oxidative Stress and Mitochondrial Complex I Dysfunction Correlate with Neurodegeneration in an α-Synucleinopathy Animal Model
Source: Int J Mol Sci. 2022 Sep 27;23(19):11394. doi: 10.3390/ijms231911394 (PMC9570254; doi:10.3390/ijms231911394)
Supplement: Supplementary file 1 [file ijms-23-11394-s001.zip › ijms-1860398-supplementary.pdf]

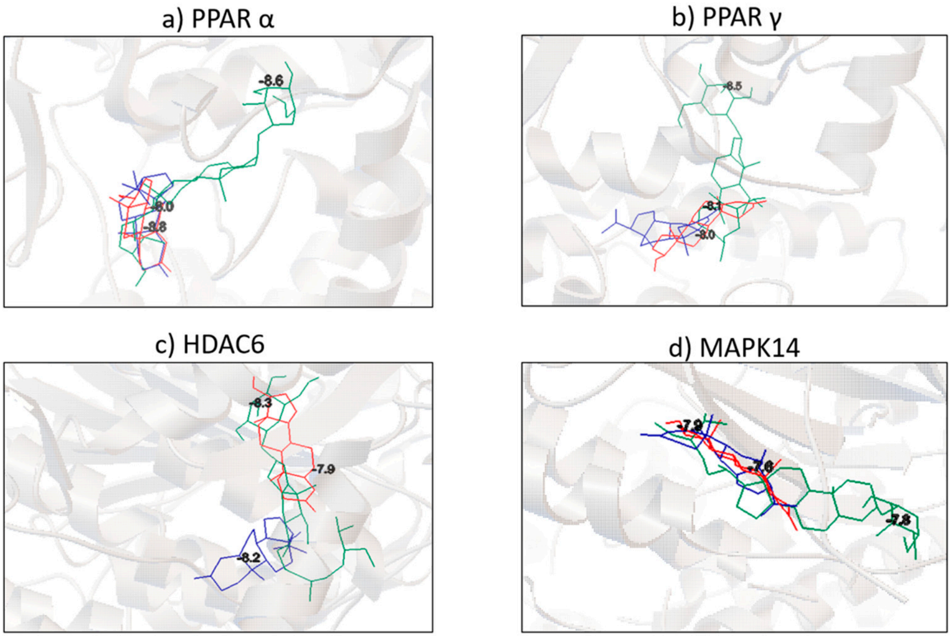

Figure S1. Molecular docking 3D representations between ligands (BSSG and positive controls) and (**a**) PPARα, (**b**) PPARγ, (**c**) HDAC6 and (**d**) MAPK14. BSSG is represented in green, whereas positive controls are represented in red (testosterone) and blue (progesterone). These 3D representations were obtained from AutoDock tools.

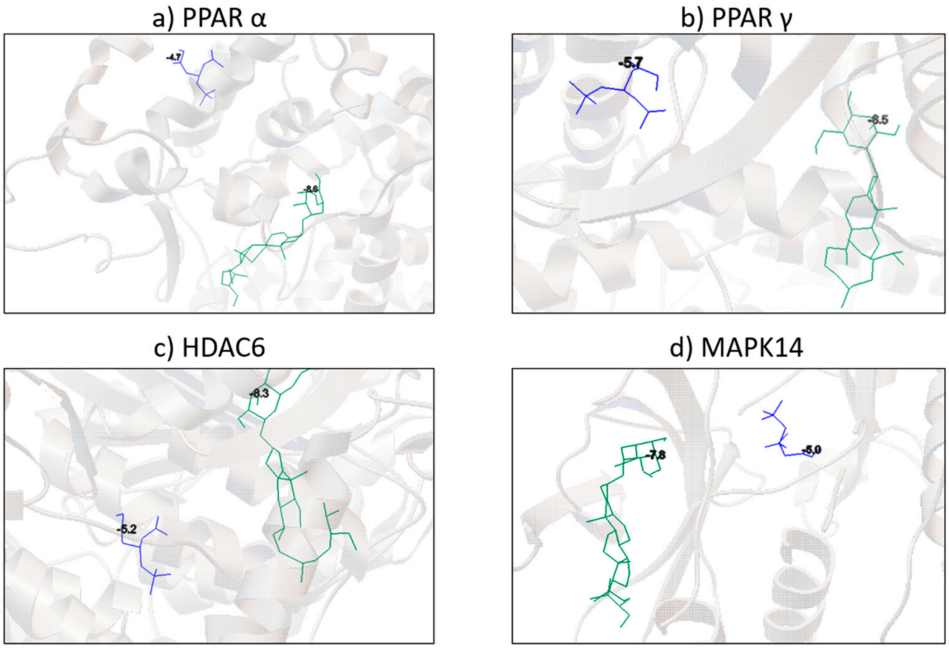

Figure S2. Molecular docking 3D representations between ligands (BSSG and negative control) and (**a**) PPARα, (**b**) PPARγ, (**c**) HDAC6 and (**d**) MAPK14. BSSG is represented in green, whereas the negative control (propanaminium) is in blue. These 3D representations were obtained from AutoDock tools.

Table S1. Comparative results of molecular docking between BSSG and negative (-) and positive (+) controls.

Compound	ΔG (kcal/mol)			
	PPAR α	PPAR γ	HDAC6	MAPK14
Propanaminium (-)	-4.7	-5.7	-5.2	-5.0
Testosterone (+)	-8.0	-8.1	-7.9	-7.6
Progesterone (+)	-8.8	-8.0	-8.2	-7.9
BSSG	-8.6	-8.5	-8.3	-7.8

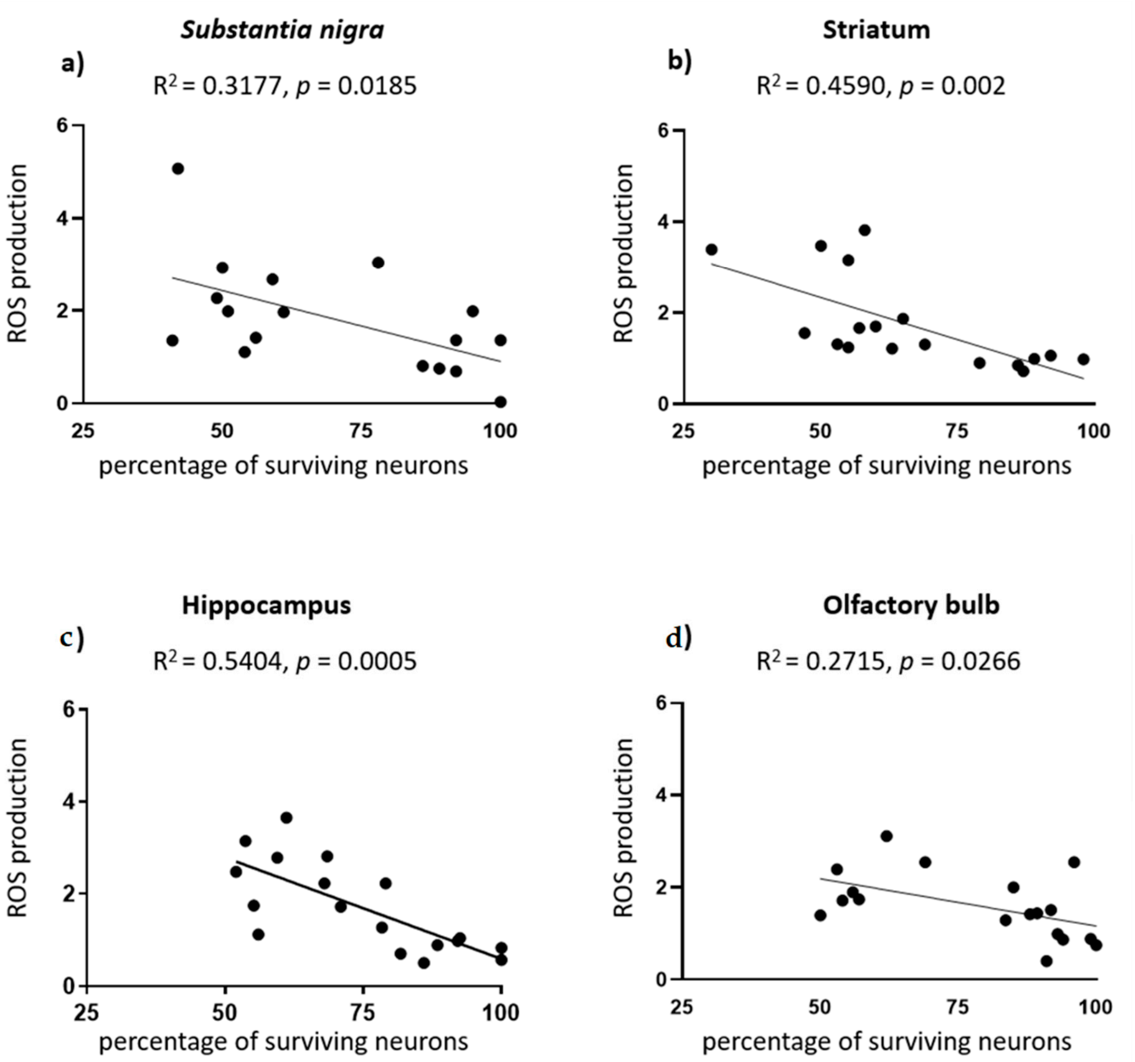

Figure S3. Correlation analysis of radical oxygen species (ROS) with the percentage of survival neurons in *substantia nigra* (**a**), striatum (**b**), hippocampus (**c**), and olfactory bulb (**d**). Pearson's correlation coefficient and linear regression appear on the top of every graph. $p < 0.05$ was considered a statistically significant difference.

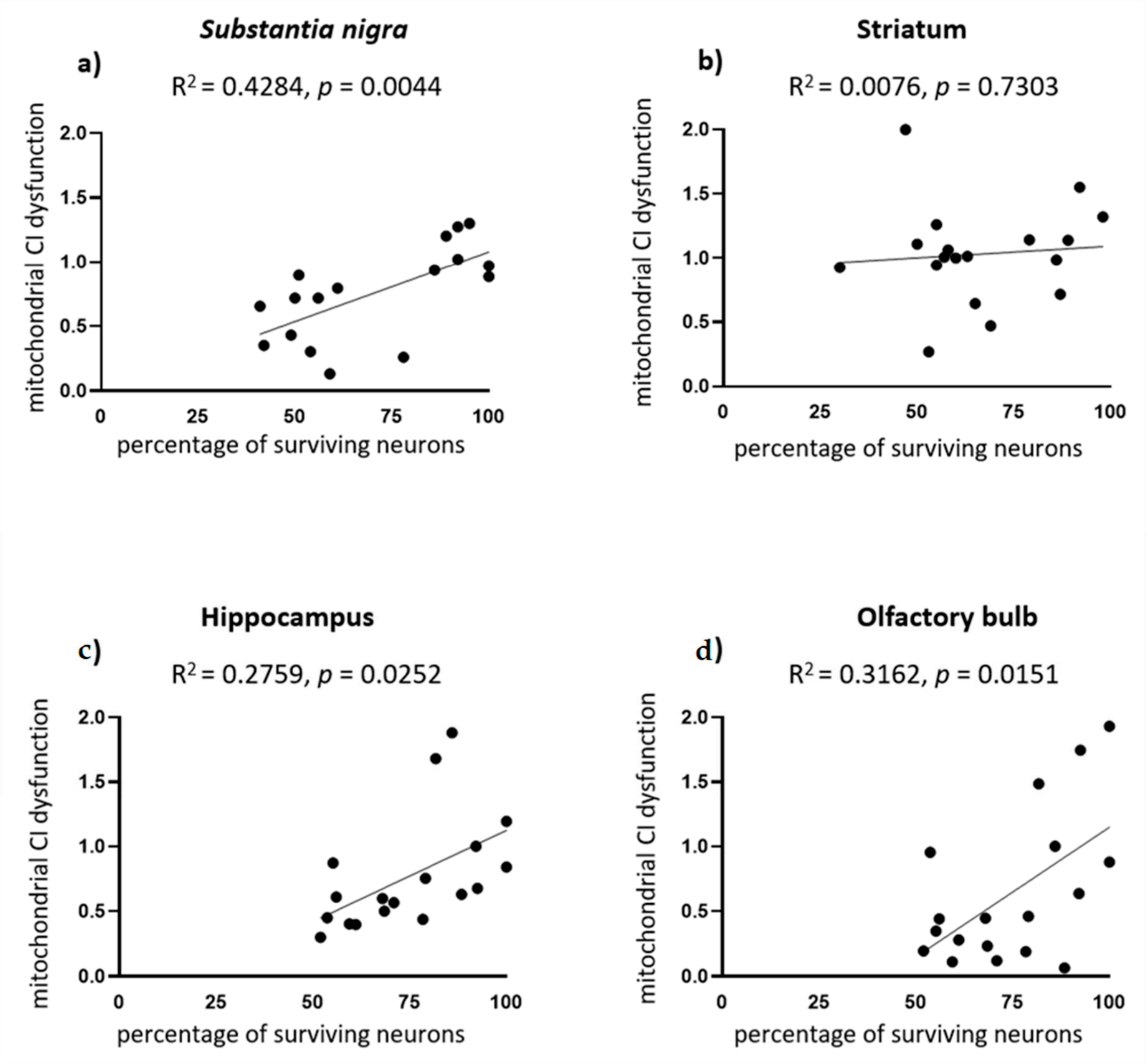

Figure S4. Correlation analysis of mitochondrial complex I (CI) dysfunction with the percentage of survival neurons in *substantia nigra* (**a**), striatum (**b**), hippocampus (**c**), and olfactory bulb (**d**). Pearson's correlation coefficient and linear regression appear on the top of every graph. $p < 0.05$ was considered a statistically significant difference.

a)

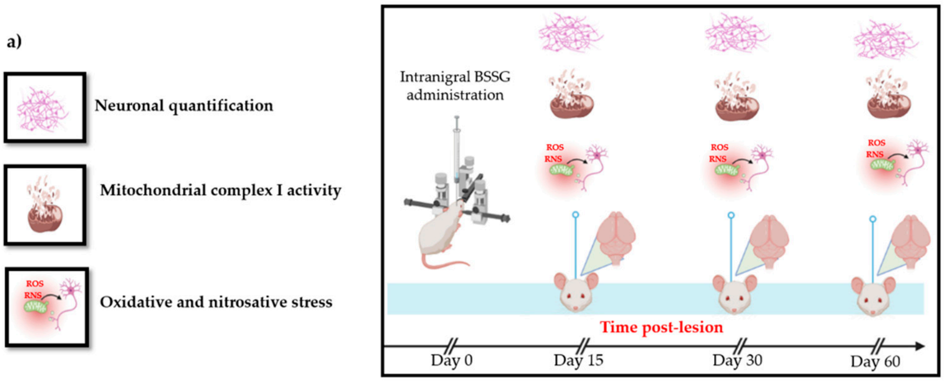

b)

Experimental groups	Experimental assays		
	Oxidative and nitrosative stress	Mitochondrial complex I activity	Neuronal quantification
Untreated	*n* = 3 x 2*	*n* = 3 x 2*	*n* = 6
Mock	*n* = 3 x 2*	*n* = 3 x 2*	*n* = 6
BSSG 15 days	*n* = 3 x 2*	*n* = 3 x 2*	*n* = 6
BSSG 30 days	*n* = 3 x 2*	*n* = 3 x 2*	*n* = 6
BSSG 60 days	*n* = 3 x 2*	*n* = 3 x 2*	*n* = 6
Number of animals per experiment	*n* (15 x 2*) = 30	*n* (15 x 2*) = 30	*n* = 30
Total number of animals per group and experiment (*n* = 90)			

Figure S5. Experimental design. (**a**) Representative illustration of experimental assays evaluated through a temporal line. (**b**) The table shows the number of animals used by subgroups and experimental assays. * Since *n* = 6 animals per GROUP were used in the biochemical assays, these were performed in two independent experiments (*n* = 3 animals per group and experiment). The Figure was created using Biorender.com.
